# Diagnostic utility of acute phase proteins and their ability to guide antibiotic usage in pigs, horses, and cattle: a mapping review

**DOI:** 10.1186/s13028-024-00766-6

**Published:** 2024-09-05

**Authors:** Nadia Jakobsen, Nicolai Rosager Weber, Inge Larsen, Ken Steen Pedersen

**Affiliations:** 1 Department of Veterinary and Animal Sciences, Faculty of Health and Medical Sciences, University of Copenhagen, Grønnegårdsvej 2, 1870 Frederiksberg C, Denmark; 2 Danish Agriculture & Food Council F.M.B.A, Axeltorv 3, 1609 Copenhagen, Denmark; 3 Ø-Vet A/S, Køberupvej 33, 4700 Næstved, Denmark

**Keywords:** Antimicrobial use, Diagnostics, Infectious disease, Veterinary medicine

## Abstract

To mitigate the use of antibiotics for many of the multifactorial diseases seen in pigs, horses and cattle, new diagnostic tools are needed. Acute phase protein (APP) measurements can, in humans, be used to guide antibiotic treatment initiation, evaluate treatment efficacy, and make a prognosis. The aim of this review is to collect evidence on the clinical functionality of APP measurements as a tool to guide antibiotic treatment in pigs, horses, and cattle. Literature was retrieved using Medline, CAB Abstracts and Google Scholar. The acute phase response has been investigated for a plethora of diseases and clinical signs and the major acute phase proteins are elevated in diseased compared to healthy animals. Few studies correlated acute phase response with aetiology, antibiotic treatment efficacy, prognosis, or severity of disease. The existing research does not support that APP can be used to guide antibiotic treatment, but the reported studies indicate that C-reactive protein (CRP) might be able to differentiate between bacterial and non-bacterial causes of disease in pigs. Serum amyloid A (SAA) might reflect underlying aetiology in horses and infectious or non-infectious cases of mastitis in cows.

## Background

One challenge in large animal medicine regarding prudent use of antibiotics is that clinical signs alone cannot be used to determine underlying aetiology. Examples are respiratory disease with both bacterial and viral aetiologies [1], mastitis where some cases will self-resolve [2] and lameness with both infectious and traumatic causes [3]. Among the diagnostic tools are post-mortem examinations and laboratory testing e.g. PCR, bacterial cultivation and somatic cell count [1, 2, 4], which can be laboursome and expensive. Further, these procedures may not be compatible with the need to promptly decide if antibiotic treatment is necessary when confronted with one or more sick animals [5, 6]. Consequently, veterinarians are at risk of using and prescribing antibiotics based on empirical diagnosis and without identifying the causative agent [5], which can lead to overuse of antibiotics and further increase the risk of antimicrobial resistance [7, 8].

Acute phase proteins (APPs) are key elements of the innate immune system and are a part of the acute phase response (APR). The acute phase proteins are mainly synthesized by hepatocytes and the production is cytokine dependent. Interleukin-6 (IL-6), interleukin 1 (IL-1) and tumor necrosis factor-alpha (TNF-α) are major inducers [9, 10]. Generally, the response is unspecific and connected to both infection, trauma or inflammation [11]. However, other conditions such as heat stress [12], parturition [13, 14] and intensive training [15, 16] have been associated with altered APP levels in animals.

C-reactive protein (CRP) and serum amyloid A (SAA) are used in humans to differentiate between bacterial and non-bacterial causes of disease, and to guide initiation of antibiotic treatment, thus potentially reducing unnecessary antibiotic use [17, 18]. Additionally, CRP could reduce antibiotic treatment duration in children and treatment initiation in adults without adverse negative effects [19, 20]. C-reactive protein differentiate infectious from non-infectious aetiologies which could prevent unnecessary antibiotic initiation in diseases such as systemic inflammatory response syndrome [20], respiratory disease [19, 21], diagnosis of septic arthritis and osteomyelitis [22] and unspecific fever [23, 24]. The widespread use of APPs to optimize antibiotic use in humans, as well as a recently published review on biomarkers and sepsis in animals [25], suggests that APPs could be a valuable tool in veterinary medicine.

The acute phase response is species and disease dependent. This means that the magnitude of the response and which APPs are affected differ between species and conditions [10, 11]. Therefore, knowledge of the APR in specific species and diseases is needed to establish the clinical utility of APPs for guiding antibiotic use in veterinary medicine. The aim of this mapping review was therefore to investigate existing knowledge on APPs in pigs, horses and cattle and the potential to optimize antibiotic treatment for diseases in these animals.

### Search strategy

Relevant literature on APPs and diagnostic use of APPs was retrieved by searching Medline (1946 to March 2023), Google scholar and CAB Abstracts (1973–March 2023). Literature published up until March 2023 was considered. Abstracts were read, and relevant literature retrieved. Duplicates and studies that were not peer-reviewed or in English were excluded. Search terms used were: “Acute phase”, “Antimicrobial*”, “Antibiotic*”, “Cow”, “Pig” and “Horse”.

### Review

#### Pigs

The porcine APR has been studied in experimentally and naturally infected pigs and in pigs with clinical signs of disease or injury. In growing pigs, the APR has been studied for a plethora of experimentally induced bacterial and viral diseases either alone or as co-infections. Examples are *Streptococcus suis* [26]*,* porcine reproductive and respiratory syndrome (PRRS) [27, 28], *Actinobacillus pleuropneumoniae* (AP) [29]*,* swine influenza and *Pasteurella multocida* [30, 31], *Bordetella bronchiseptica* [32], *Pasteurella multocida* [33]*,* swine influenza and *Bordetella bronchiseptica* [34, 35] and swine influenza and *Actinobacillus pleuropneumoniae* [36]*.* In finishing pigs, the studies have focused on *Actinobacillus pleuropneumoniae* [37–39], *Streptococcus suis* [40], osteomyelitis [41], African swine fever (ASF) and Aujeszky’s disease (AD) [42]. In sows, experimentally induced infection of the mammary gland with *E. coli* has also been investigated [43]. Information regarding induced diseases, affected APPs and age groups has been summarized in Table 1.

**Table 1:** Overview of studies on acute phase protein (APP) levels in pigs for experimentally induced diseases^1^

Reference	Age group^2^	Disease^3^	Investigated APPs^4^
[36]	GP	Swine influenza (H1N1) AP H1N1 + AP	SAA*, MAP, HP, CRP SAA*, MAP*, HP, CRP* SAA*, MAP*, HP, CRP*
[35]	GP	Swine influenza (H1N1) + *B. bronchiseptica*	SAA* (40-fold increase), MAP* (Threefold increase), HP* (Fivefold increase), CRP* (Eightfold increase)
[31]	GP	Swine influenza (H1N1) + *P. multocida*	SAA* (40-fold increase), MAP* (Fourfold increase), HP* (Fourfold increase), CRP* (Eightfold increase)
[30]	GP	Swine influenza (H3N2) + *P. multocida*	SAA* (50-fold increase), MAP* (Threefold increase), HP* (Sixfold increase), CRP* (Fivefold increase)
[34]	GP	Swine influenza (H3N2) + *B. bronchiseptica*	SAA* (30-fold increase), MAP* (Threefold increase), HP* (Sevenfold increase), CRP* (Eightfold increase)
[33]	GP	*P. multocida*	SAA* (30-fold increase), MAP* (Fourfold increase), HP* (Fivefold increase), CRP* (Eightfold increase)
[32]	GP	*B. bronchiseptica*	SAA* (30-fold increase), MAP* (Threefold increase), HP* (Fivefold increase), CRP* (Eightfold increase), AGP
[28]	GP	PRRS	CRP*, HP* (both saliva and serum)
[27]	GP	PRRS	AGP, HP*
[26]	GP	*S. suis*	SAA* (40-fold increase), Pig-MAP* (Sevenfold increase), HP* (Tenfold increase), CRP* (Tenfold increase), Apo A-I* (40% reduction), Alb
[39]	FP	AP	CRP*, HP*
[37]	FP	AP	CRP* (Sevenfold increase), HP* (40-fold increase), MAP* (12—fold increase)
[38]	FP	AP	CRP* (13-fold increase), HP, SAA* (89-fold increase),
[42]	FP	ASF ADV	MAP*, CRP*, HP*, Alb MAP*, CRP*, HP*, Alb
[40]	FP	*S. suis*	HP* (12-fold increase)
[41]	FP	Osteomyelitis (*S. aureus*)	HP*, CRP*, SAA
[43]	S	Intramammary inoculation of E. *coli*	SAA* (Tenfold increase), HP

^1^Acute phase proteins marked with * differ significantly pre and post infection (P < 0.05)

^2^*GP* Growing pigs (weaning to 10 weeks of age), *FP* Finishing pigs (10 weeks—slaughter), S = Sows (1 + parities)

^3^*AP Actinobacillus pleuropneumoniae*, *PRRS* Porcine respiratory and respiratory syndrome, *ASF* African swine fever, *ADV* Aujeszky’s disease

^4^*CRP* C-reactive protein (μg/mL), *SAA* Serum Amyloid A (μg/mL), *HP* Haptoglobin (mg/mL), *MAP* Pig major acute phase protein (mg/mL), *Alb* Albumin (g/dL), *Apo A-I* Apo lipoprotein A-I (mg/mL)

The experimental inoculations revealed that all moderate and major APPs are indeed elevated in most diseases in the week post-infection. Exceptions are osteomyelitis (*S. suis*), intramammary inoculation of *E. coli* and swine influenza (H1N1), where SAA [41], haptoglobin (HP) [43] and CRP, pig major acute phase protein (Pig-MAP) and SAA [36] remained unchanged respectively. Regarding the elevation of HP in AP infection the results are equivocal with two studies finding no effect [36, 38] and two studies finding an effect [37, 39]. This difference could be affected by inoculation method: A viral infection comparing an aerosol to an intranasal inoculation showed that aerosol inoculation lead to a more severe pathology in the lower respiratory tract [44, 45] and induced a higher immune and cytokine response [45]. Furthermore, one of the studies with no effect followed the pigs for 18–24 h post infection [38] which might be too short a time period to detect a HP response, since HP have been shown to have a delayed response in pigs [46]. In pigherds several infections can occur simoultaneosly and evidence on the APP response in bacterial and co-infections suggests that being co-infected with viral and bacterial pathogens should not mask a bacterial infection [30–35]. However, in order to distinguish bacterial from viral infections a significant difference between APP levels is needed so that a cut-off level with high sensitivity and specificity can be established. The studies inducing PRRS, ASF and AD did not provide the magnitude of the increase or the mean APP levels pre- and post-infection. Therefore, approximations were made based on the illustrations provided. The ranges are included in Table 2. Minding the decreased reliability, CRP levels seemed lower in viral infections compared to bacterial infections. Additionally, CRP but not HP, followed the reversion of clinical signs in pigs treated with danofloxacin and hence CRP might be used as a clinical evaluation of treatment efficacy [39].

**Table 2:** Increase in acute phase proteins in experimentally induced bacterial and viral infections in pigs

	HP	CRP	MAP
Viral^1^	3–12 fold	3–Fivefold	Sixfold
Bacterial	5–40 fold	7–13 fold	3–12 fold

^1^The magnitude of the increase in viral infections was approximated based on illustrations from Asai et al., [27] Carpintero et al., [42], and Gómez-Laguna et al. [28],

^2^*CRP* C-reactive protein (μg/mL), *SAA* Serum Amyloid A (μg/mL), *HP* Haptoglobin (mg/mL), *MAP* Pig major acute phase protein (mg/mL)

In experimentally induced infections the exact time of inoculation is known, and blood samples can be taken repeatedly to study the kinetics and establish maximum APP levels. However, in practice most infections are continously circulating in the herds and pigs might be chronically infected or several days into the acute phase before diagnostics are performed. The half-life of CRP is 47 h with elevations seen 6 h post-stimulus, for SAA the half-life is 35 h and for HP it is 3–5 days [25]. In addition, studies have shown that Pig-MAP increases 2 days post-infection and reaches normal levels within 5 to 10 days post-infection [32, 36]. Therefore, if blood samples are not performed within the first days post-infection, the CRP and SAA levels migth already have decreased below physiological levels. In addition, the pigs might suffer from a mixture of infections and injuries, which might blur the response. Hence in order to conclude if APPs can be used for antibiotic guidance on farm, trials with naturally occurring diseases and clinical signs are needed. One such study investigated the correlation between HP levels and different clinical signs of disease in seven specific pathogen free (SPF) herds and four non-declared herds [47]. Specific pathogen free is a Danish health system which is used to declare the health status of a herd regarding seven specific pathogens. The targeted diseases are enzootic pneumonia, porcine pleuropneumonia, porcine reproductive and respiratory syndrome, swine dysentery, atrophic rhinitis, mange and lice [48]. The study focused on respiratory disease, atrophic rhinitis, lameness, tail or ear bite, diarrhoea, umbilical hernia and neurological disease in finishing pigs. The study found significantly increased HP levels in pigs with clinical signs of lameness and tail or ear bite (P < 0.001). However, no difference was seen in pigs with respiratory signs or umbilical hernias. Diarrhoea, acute rhinitis and neurological signs were excluded due to low prevalence. Additionally, the study found a herd effect and an interaction between the herd health status (SPF vs. non-declared) and the age of the finishing pigs (P < 0.05) [47]. An effect of herd health status on acute phase protein levels has been shown in another study [49] indicating that baseline APP levels can differ between herds with differing health status, which should be considered when interpreting the magnitude of the APR.

As can be seen from Table 3, significant increases in one or more APPs were found for all diseases. C-reactive protein and SAA were elevated in all diseases but AD, HP in all diseases, Pig-MAP was elevated in Porcine circovirus type 2 (PCV2), *M. hyopneumoniae* and inflammation and albumin (Alb) was significantly decreased in PRRS, PCV2 and inflammation. Interestingly, and in line with findings from experimentally induced infections, CRP was the only APP where a larger response was apparent in bacterial disease (71-fold increase), compared to viral (9 and 26-fold increase) and inflammation (38-fold). Indicating that CRP might, as seen in humans [18, 50], be a candidate for antibiotic guidance in pigs.

**Table 3:** Overview of acute phase protein (APP) levels in pigs for common diseases and clinical signs^1^

Reference	Age group^2^	Disease^3^	APP levels diseased pigs^4^	APP levels healthy pigs^4^
[47]	FP	Lameness	HP: 8.13^a^	HP: 1.48^b^
		Tail or ear bite	HP: 25.7^a^	HP: 0.47^b^
[49]	GP/FP	PRRS	CRP: 47.96^a^ SAA: 7.36^a^ HP: 1.41^a^ MAP: 1.05^a^ Alb: 2.79^b^	CRP: 5.32^b^ SAA: 3.10^b^ HP: 0.21^b^ MAP: 0.76^a^ Alb: 3.21^a^
		AD	CRP: 12.19^a^ SAA: 0.27^a^ HP: 1.81^a^ MAP: 1.08^a^ Alb:3.35^a^	CRP: 5.32^a^ SAA: 3.10^a^ HP: 0.21^b^ MAP: 0.76^a^ Alb: 3.21^a^
		PCV2	CRP: 139.04^a^ SAA: 72.4^a^ HP: 5.03^a^ MAP: 3.32^a^ Alb: 2.48^b^	CRP: 5.32^b^ SAA: 3.10^b^ HP: 0.21^b^ MAP: 0.76^b^ Alb: 3.21^a^
		*M. hyopneumoniae*	CRP: 380.95^a^ SAA: 16.24^a^ HP: 3.5^a^ MAP: 2.16^a^ Alb: 3.24^a^	CRP: 5.32^b^ SAA: 3.10^b^ HP: 0.21^b^ MAP: 0.76^b^ Alb: 3.21^a^
		Inflammation	CRP: 203.15^a^ SAA: 26.05^a^ HP: 4.06^a^ MAP: 2.55^a^ Alb: 2.82^b^	CRP: 5.32^b^ SAA: 3.10^b^ HP: 0.21^b^ MAP: 0.76^b^ Alb: 3.21^a^
[51]	FP	Diarrhoea	HP: 1.42^a^	HP: 0.76^b^
		Lameness	HP: 2.19^a^	HP: 0.74^b^
		Respiratory signs	HP: 1.41^a^	HP: 0.90^b^
		Tail or ear bites	HP: 1.99^a^	HP: 0.91^b^
[114]	GP	Culled pigs	HP: 2.23^a^ CRP: 252.9^a^	HP: 1.42^b^ CRP: 84.88^b^
[52]	SP	SINS	**HP: 82.8/69.7/51.9**^**5**^ **CRP: 1.39/2,21/3.16** Fb: 105/113/112	
	GP		HP: 88.6/107.0/125.5 CRP: 10.99/11.48/11.85 Fb: 166/171/177	
	FP		HP: 416.0/370.4/491.8 **CRP: 11.15/14.24/15.75** **Fb: 187, 293, 333**	

^1^Means (medians in [49])—columns with different superscripts differ significantly (P < 0.05)

^2^*SP* Suckling Pigs (Farrowing to weaning), *GP* Growing pigs (weaning to 10 weeks of age), *FP* Finishing pigs (10 weeks to slaughter)

^3^*PRRS* Porcine respiratory and respiratory syndrome, *AD* Aujeszky’s disease, *PCV2* Porcine circovirus type 2, *SINS* swine inflammation and necrosis syndrome

^4^*CRP* C-reactive protein (μg/mL), *SAA* Serum Amyloid A (μg/mL), *HP* Haptoglobin (mg/mL), *MAP* Pig Major acute phase protein (mg/mL), *Alb* Albumin (g/dL), *Fb* Fibrinogen (mg/dL)

^5^Comparison in APP between SINS scores (Low/medium/high)—values in bold are significantly correlated to score of SINS

The mean APP levels of three other studies performed on suckling, growing and finishing pigs has been summarized in Table 3. In accordance with the other studies, significant differences between pigs with and without clinical signs were discovered [46, 50] although few discrepancies exist e.g. in respiratory disease which altered a non-significant [47] and a significant increase in HP [51]. This could be due to differences in aetiology or subclinical disease, which would also explain the high HP levels in the control group [47]. The third study, which investigated the correlation between swine inflammation and necrosis syndrome (SINS) score and APP levels, found that for suckling pigs HP and CRP correlated with SINS score, and for fattening pigs CRP and fibrinogen (Fb) correlated with SINS score [52]. Studies investigating the same porcine diseases are lacking, hence more research is needed to conclude which APPs can be used diagnostically for which diseases. However, Receiver-operating-characteristic curve (ROC-curve) analysis was used to establish the accuracy of using HP as an objective marker of clinical disease and an accuracy of 76–78% was found for lameness, diarrhoea and tail or ear bites and an accuracy of 67% was found for respiratory disease [51].

In sows, the majority of studies on APP investigated postpartum dysgalactia syndrome (PDS), a multifactorial syndrome causing decreased growth and increased mortality in piglets due to dysgalactia, fever, mastitis and/or metritis in affected sows [53]. However, one study investigated the CRP and HP response in vulvar discharge syndrome (VDS) and found no significant differences in sows with and without VDS [54]. The effect of reproductive disease on APP levels has been summarized in Table 4. The table contains data from sows with PDS and VDS, but only days where significant differences between groups were found, were included. Around parturition an APP dependent increase in levels occurs, with healthy sows showing lower rises than PDS sows [13, 14, 43]. For instance, a study found that HP, presented with two peaks around parturition in both healthy and PDS sows and that the HP levels were significantly elevated from day −14 to + 14 days postpartum [14]. For CRP, Pig-MAP and SAA a peak was seen postpartum in healthy and PDS sows, with levels elevated from farrowing and until 7–14 days postpartum [14]. The elevations seen in apparently healthy sows indicate that interpreting APP levels on sows  ± 14 days of farrowing should be done with care, since rises are not necessarily indicative of underlying inflammation or disease. Three out of four studies on PDS showed significant differences between healthy and PDS sows [13, 14, 55]. The test period and investigated APPs vary considerably between studies, but rises in CRP, HP, SAA and alpha 1-acid glycoprotein (AGP) could be indicative of PDS. However, to be clinically relevant, the alterations need to be apparent either before farrowing or within the first days after farrowing and be correlated to clinical signs of PDS. Serum amyloid A levels has been shown to be correlated (R-spearman—0.76) with PDS before farrowing. The study found sensitivity, specificity and kappa-value of 0.91, 0.99 and 0.92, respectively, using a threshhold of 0.63 [14]. However, no explanation of the clinical exam used as a gold standard for PDS sows, the inclusion and exclusion criteria in all groups and the sampling procedure was provided in the paper, making it difficult to interpret if the sows included were representative of parturating sows in general.

**Table 4:** Overview of acute phase protein (APP) levels in sows and gilts for naturally occurring diseases^1^

Reference	Age group^2^	Disease^3^	Test period	APP levels diseased pigs^4^	APP levels healthy pigs^4^
[14]	S	PDS	Day -28 to 28^*^	CRP d 7: 155^a^ HP d 7: 2.1^a^ SAA d -7: 100^a^ SAA d -3: 40^a^ SAA d -1: 35^a^ SAA d 0: 35^a^ SAA d 3: 290^a^ SAA d 7: 90^a^ MAP d -7: 2.2^a^ MAP d -3: 2.2^a^ MAP d 7: 3.1^a^ MAP d 14: 2.2^a^	CRP d 7: 60^b^ HP d 7: 0.9^b^ SAA d -7: 20^b^ SAA d -3: 10^b^ SAA d -1: 5^b^ SAA d 0: 5^b^ SAA d 3: 20^b^ SAA d 7: 5^b^ MAP d -7: 1.2^b^ MAP d -3: 1.05^b^ MAP d 7: 1.7^b^ MAP d 14: 1.0^b^
[13]	S	PDS	Day – 3 to 2 (every 24 h)	HP d 1: 2.73^a^ HP d 2: 2.92^a^	HP d 1: 2.46^b^ HP d 2: 2.56^b^
[55]	S	MMA	Day 1 to 20 (every 5th day)	AGP d 10: 628.5^b^ AGP d 20: 519.4^b^ HP d 1: 0.21^a^ HP d 5: 0.21^a^ HP d 10: 0.40^a^	AGP d 10: 869.1^a^ AGP d 20: 429.3^a^ HP d 1: 0.02^b^ HP d 5: 0.04^b^ HP d 10: 0.28^b^
[54]	S	VDS	-	HP: 2.5^a^ CRP: 30.3^a^	HP: 2.3^b^ CRP: 25.9^b^

^1^Means—columns with different superscripts differ significantly (P < 0.05)

^2^*G* Gilts (First parity), *S* Sows (1 + parities)

^3^*PDS* postpartum dysgalactica syndrome, *MMA* Mastitis, metritis and agalactica

^4^*CRP* C-reactive protein (μg/mL), *SAA* Serum Amyloid A (μg/mL), *HP* Haptoglobin (mg/mL), *MAP* Pig Major acute phase protein (mg/mL), *AGP* Alpha 1-acid glycoprotein (μg/mL)

^*^Test performed on day −28, −14, −7, −3, −1, 0, 1, 3, 7, 14 and 28

#### Summary—pigs

The moderate to major APPs in pigs are CRP, SAA, HP, and Pig-MAP, which all show a large response in most of the diseases or syndromes studied. An acute phase response was elicited by all experimental and naturally occurring bacterial and viral diseases that are common in intensive pig production. No studies compared APP levels for viral or bacterial causes of the same clinical sign, so approximations were made based on the limited literature investigating diseases with different aetiologies. C-reactive protein appears to alter a larger APR in bacterial disease than in viral disease and as a result, CRP may be a candidate for guiding antibiotic initiation in pigs and evaluate treatment efficacy, though additional research is required to confirm this. Furthermore, the interpretation depends on the magnitude of the increase and thus it should be noted that parturition, age, and general health status influence baseline levels.

#### Horses

Compared to pigs, not many studies were found investigating experimentally induced diseases in horses. However, one study induced ascending placentitis in mares by intracervical inoculation with *Streptococcus equi* [56]. As can be seen from Table 5, both SAA and HP were significantly increased whereas Fb was not.

**Table 5:** Overview of acute phase protein (APP) levels in mares for experimentally induced diseases^1^

Reference	Type	Age group^2^	Disease	Investigated APPs^3^
[56]	Exp	H	Ascending placentitis	SAA*, HP*, Fb

^1^Acute phase proteins marked with * differ significantly pre and post infection (P < 0.05)

^2^*H* Adult horses

^3^*SAA* Serum Amyloid A (μg/mL), *HP* Haptoglobin (mg/mL), *Fb* Fibrinogen (mg/dL)

Several naturally occurring diseases were investigated e.g. joint disease [57, 58], respiratory disease [59, 60], intestinal disease [59, 61], infection with *L. interrogans* [62] and parasitic disease [63–65]. The investigated APPs and obtained levels in diseased and non-diseased horses have been summarized in Table 6. All studies investigated SAA, which is the major acute phase protein in horses, but data on alterations in Alb, Fb, HP and CRP was also found. Several of the studies determined aetiology and APP responses in infectious and non-infectious causes of disease. For septic arthritis, significantly higher SAA levels were found in horses with bacterial infections compared to culture negative horses, and levels in fungal infections were in between [58]. In a study on joint diseases e.g. osteoarthritis, osteochondrosis, infectious arthritis and infectious tenovaginitis, significant differences were found between healthy and infectious aetiologies. No difference was shown between infectious and non-infectious causes of disease although large numerical differences were seen [66], perhaps due to low sample sizes in the infectious and non-infectious group respectively (n = 7 and 9) [66]. In foals presented with weakness, pneumonia or diarrhoea, significant differences in SAA and Fb were seen between bacterial and non-bacterial infections, indicating that SAA might for some disease complexes be suitable to monitor antibiotic usage in horses although more research is needed to cooperate the findings.

**Table 6:** Overview of acute phase protein (APP) levels in horses for common diseases and clinical signs^1^

Reference	Age group^2^	Disease	APP levels diseased horses^3^	APP levels healthy horses^3^
[58]	H	Septic arthritis	Gram positive: SAA: 3200^b^ Fb: 410^a^ CRP: 177^a^ Gram negative: SAA: 3800^b^ Fb: 535^a^ CRP: 97^a^ Fungi: SAA: 2500^ab^ Fb: 620^a^ CRP: 94^a^	Culture negative: SAA: 1100^a^ Fb: 485^a^ CRP: 94^a^
[66]	H	Joint disease	Infectious: SAA: NA^b^ Non-infectious: SAA: NA^ab^	Healthy: SAA: NA^a^
[59]	F	Pneumonia, weakness and diarrhoea	Bacterial: SAA: 65^a^ Fb: 660^a^ Non-bacterial: SAA: 1.6^b^ Fb: 350^b^	
[64]	H	*Theileria equi*	Pasture: SAA:0.48^a^ HP: 20.41^a^ CRP: 36.3^a^ Fb: 400^a^ Stabled: SAA: 0.48^a^ HP: 18.3^a^ CRP: 33.8^a^ Fb: 300^a^	Pasture: SAA:0.48^a^ HP:21.28^a^ CRP: 25.8^a^ Fb: 300^a^ Stabled: SAA: 0.48^a^ HP: 13.1^a^ CRP: 25.4^a^ Fb: 300^a^
[60]	H	Inflammatory airway diseases	SAA: NA^a^ HP: NA^a^	SAA: NA^b^ HP: NA^b^
[63]	H	Habronemosis	SAA: 16.36^a^ HP: 1.3^a^	SAA: 10.45^b^ HP: 0.73^b^
[65]	H	Protozoal myeloencephalitis	SAA: < 0.1^a^ CRP: < 0.1^a^	SAA: < 0.1^a^ CRP: < 0.1^a^
[62]	H	*L. interrogans*	SAA: 14.1^a^ HP: 5.3^a^ Alb: 0.298^a^	SAA: 5.85^b^ HP: 1.26^b^ Alb: 0.305^a^
[61]	F	Proliferative enteropathy	SAA: 466^a^	SAA: 192^a^

^1^medians—columns with different superscripts differ significantly (P < 0.05)

^2^*F* Foals, *H* Adult horses

^3^*CRP* C-reactive protein (μg/mL), *SAA* Serum Amyloid A (μg/mL), *HP* Haptoglobin (mg/mL), *Fb* Fibrinogen (mg/dL), *Alb* Albumin (g/dL)

The clinical relevance of APPs was investigated in two studies [60, 63]. Combining surfactant protein D and SAA and a cut-off of 0.80 (Log) for the sum, inflammatory airway disease (IAD) and control horses could be distinguished with a sensitivity and specificity of 100% and 90%, respectively. Furthermore, surfactant protein D and HP rendered a sensitivity and a specifictiy of 100%, when using a cut-off of 7.70 (Log) [60]. The diagnostic potential of HP and SAA in the parasitic disease habronemosis has been studied. The study found that using a threshold of 0.85 and 11.66 for HP and SAA provided a sensitivity, specificity, and accuracy of 87%/90%, 81%/86% and 87%/94%, respectively [63]. Using HP would correctly classify 84.3% of the horses and for SAA, this would be true for 88.2%. That APP levels can be used to find parasitic infections is ambiguous since no difference in APP levels was found in protozoal myeloencephalitis and *Theileria equi* infections [64, 65]. Emphasizing that a one-size-fits-all solution, is not possible regarding the diagnostic and prognostic value of APP levels and that more research is needed before APP levels can be used to guide antimicrobial treatment.

#### Summary—horses

The major acute phase protein in horses is SAA, but CRP, HP and Fb are frequently studied concurrently. In most of the investigated diseases or syndromes SAA, CRP and HP were significantly elevated in diseased versus non-diseased horses, except in parasitic disease where evidence suggests a lack of response. Regarding the use of APP levels to differentiate between infectious and non-infectious causes of disease, more studies have been carried out for horses than pigs, and although solid evidence is still lacking, SAA could be a candidate.

#### Cattle

For cattle, the most investigated disease is mastitis, both in its clinical and subclinical form as well as when experimentally induced. Studies involving experimentally induced mastitis focused mainly on *E. coli* mastitis, and the relevant APPs have been summarized in Table 7. As with naturally occurring mastitis, the main APPs in focus were serum and milk HP and amyloid A. However, albumin was also studied [67]*.* In serum, both SAA and HP were found to be increased significantly in *E. coli* and *S. aureus* infections [68–70]. Milk HP was not significantly increased after inoculation with *S. aureus* [69]. The increase in SAA and HP peaked around 2–5 days after inoculation [67–70] and the pattern was comparable in milk and serum samples [69].

**Table 7:** Overview of acute phase protein (APP) levels in cows for experimentally induced diseases^1^

Reference	Age group^1^	Disease	Investigated APPs^3^
[67]	C	Mastitis (*E. coli*)	Alb*
[68]	C	Mastitis (*E. coli*)	SAA*
[69]	C	Mastitis (*S. aureus*)	Serum: HP*, SAA* Milk: HP, MAA*
[70]	C	Mastitis (*E. coli*)	SAA*, HP*
[71]	C	Bovine besnoitiosis	HP*, Alb*

^1^Acute phase proteins marked with * differ significantly pre and post infection (P < 0.05)

^2^*C* cow

^3^*SAA* Serum Amyloid A (μg/mL), *MAA* Milk Amyloid A (μg/mL), *HP* Haptoglobin (mg/mL), *Alb* Albumin (g/dL)

Calves experimentally induced with bovine besnoitiosis experienced significant alterations in HP and Alb from 8 days post-infection [71]. The parasite was inoculated using three different routes and the HP response was highly method dependent with subcutaneous inoculation giving a faster response than intradermal and intravenous inoculation [71]. The kinetics of SAA, milk amyloid A (MAA) and HP seem comparable to what was found in porcine HP and SAA although HP is classified as a major APP in cows and hence a faster increase and decline would be expected [72]. Suggesting that the kinetics and half-life of the APP response might be both disease and species dependent and should be considered when interpreting APP levels in all three species.

Data on naturally occurring mastitis has been summarized in Table 8, where 10 studies investigated the association between APP levels and subclinical mastitis [73–82] and four investigated the association with clinical mastitis [78, 80, 83, 84]. In subclinical mastitis the serum and MAA, HP and Fb levels were in most studies significantly increased and albumin decreased compared to healthy cows. Furthermore, if more udder quarters were affected, a higher APP level response was seen in MAA, SAA and HP [75]. These findings suggest that APP levels can be useful in identifying cows with subclinical mastitis. The diagnostic potential of Alb, MAA, SAA serum and milk HP based on ROC-curve analysis has been summarized in Table 9. In short, the sensitivity for all APPs except Albumin was > 90% and the specificity ranged from 56.66 to 100% [73, 76, 77, 82]. The large differences in specificity could be due to variations in cut-off values and case definitions, since the studies diagnosed subclinical mastitis based on different parameters. For clinical cases, all investigated APPs were significantly altered in both milk and serum. The sensitivity and specificity based on ROC-curves are summarized in Table 9. In short, the sensitivities were between 79 and 93% and the specificities ranged from 80 to 100%. In mastitis, the APP response seems to be dependent on aetiology [85–87] with Gram-negative bacteria yielding a higher response than Gram-positive ones [85, 86]. A few exceptions exists e.g. in MAA where *A. pyogenes*, environmental S*treptococcus* and *Enterococcus* spp. yielded a high response [86, 87]. One study looked at non-infectious and infectious causes of subclinical mastitis. The data showed that cows with subclinical mastitis and positive bacteriology had higher SAA compared to cows with subclinical mastitis but negative bacteriology [82]. Additionally, cows with subclinical mastitis which resolved spontaneously had lower HP levels compared to non-self-resolving cases. Hence, HP analysis could potentially be used to identify *S. aureus* subclinical mastitis incidences not requiring antibiotic treatment [81].

**Table 8:** An overview of acute phase protein (APP) levels in cattle for naturally occurring mastitis^1^

Reference	Age group^2^	Disease	APP levels diseased cows^3^	APP levels healthy cows^3^
[73]	C	Subclinical mastitis	Serum: SAA: 50.57^a^ HP: 0.14^a^ Milk: MAA: 10.99^a^ HP: 0.17^a^	Serum: SAA: 10.99^b^ HP: 0.02^b^ Milk: MAA: 0.09^b^ HP: 0.03^b^
[74]	C	Subclinical mastitis	Milk: Fb: 28.01^a^	Milk: Fb: 10.71^b^
[75]	C	Subclinical mastitis	Milk: MAA: 24.4^b^ (1Q^4^)/ 103^a^ (2Q^4^) Serum: SAA: 25^b^ (1Q)/ 88^a^ (2Q) HP: 0.02^b^ (1Q)/ 0.42^a^ (2Q) Alb: 3.13^a^ (1Q)/ 2.98^a^ (2Q)	Milk: MAA: 1.7^c^ Serum: SAA: 17^b^ HP: 0.03^b^ Alb: 3.02^a^
[76]	C	Subclinical mastitis	Milk: MAA: 67^a^ HP: 0.039^a^ Serum: SAA:198^a^ HP: 0.17^a^	Milk: MAA: 9^b^ HP: 0.007^b^ Serum: SAA:126^b^ HP: 0.09^b^
[77]	C	Subclinical mastitis	MAA: 12.83^a^ Alb: 0.068^a^	MAA: 0.61^b^ Alb: 0.024^b^
[78]	C	Subclinical mastitis	Serum: Alb: 3.2^b^ HP: 1.89^a^ SAA: 151.61^a^ Fb: 1769^a^ Milk: HP: 0.53^a^ MAA: 22.41^a^ Fb: 789^a^	Serum: Alb: 4.3^a^ HP: 0.79^b^ SAA: 34.6^b^ Fb: 780^b^ Milk: HP: 0.061^b^ MAA: 13.6^b^ Fb: 220^b^
[79]	C	Subclinical mastitis	Serum: SAA: 2.68^a^ Milk: MAA: 0.790^a^	Serum: SAA: 2.72^a^ Milk: MAA: 0.361^b^
[80]	C	Subclinical mastitis	SAA: 47.33^a^ HP: 0.59^a^	SAA: 5.13^b^ HP: 0.09^b^
[81]	C	Subclinical mastitis	MAA: 4.79^a^ HP: 0.248^a^	MAA: 3.93^a^ HP: 0.023^b^
[82]	C	Subclinical mastitis (3 different disease groups)	Serum: SAA: 23.10/56.60/88.00^a^ Milk: MAA: 9.20/12.90/13.19^a^	Serum: SAA: 10.80^b^ Milk: MAA: 2.30^b^
[83]	C	mastitis	SAA: NA^a^ HP: NA^a^	SAA: NA^b^ HP: NA^b^
[78]	C	mastitis	Serum: Alb: 2.7^b^ HP: 2.71^a^ SAA: 189.7^a^ Fb: 1980^a^ Milk: HP: 0.99^a^ MAA: 36.19^a^ Fb: 873^a^	Serum: Alb: 4.3^a^ HP: 0.79^b^ SAA: 34.6^b^ Fb: 780^b^ Milk: HP: 0.061^b^ MAA: 13.6^b^ Fb: 220^b^
[84]	C	mastitis	Milk: MAA: 10.10^a^ HP: 216.0^a^ Serum: SAA: 118.49^a^ HP: 0.724^a^	Milk: MAA: 0.022^b^ HP: 16.20^b^ Serum: SAA: 0.69^b^ HP: 0.063^b^
[80]	C	mastitis	SAA: 129.08^a^ HP: 0.96^a^	SAA: 5.13^b^ HP: 0.09^b^

^1^means—columns with different superscripts differ significantly (P < 0.05)

^2^*Ca* Calves, *H* Heifers, *C* Cow

^3^*CRP* C-reactive protein (μg/mL), *SAA* Serum Amyloid A (μg/mL), *MAA* Milk Amyloid A (μg/mL), *HP* Haptoglobin (mg/mL), *Fb* Fibrinogen (mg/dL), *Alb* Albumin (g/dL), *AGP* Alpha 1-acid glycoprotein (μg/mL)

^4^*1Q* mastitis in 1 quarter of the udder, *2Q* mastitis in 2 quarters of the udder

**Table 9:** Receiver operating curve data calculated for mastitis using different acute-phase proteins (APPs)^1^

	Reference	APP^2^	Cut-off	Sen (%)	Spec (%)	AUC
Subclinical mastitis	[77]	Alb	0.038	88.5	86.8	0.93
	[77]	MAA	1.6	92.3	92.1	0.98
	[76]	MAA	16.4	90.6	98.3	0.99
	[73]	MAA	0.317	100	100	–
	[82]	MAA	10.0	90	56.66	0.85
	[76]	SAA	159.1	90.0	72.1	0.84
	[73]	SAA	1.99	100	100	–
	[82]	SAA	74.0	100	93.33	1.0
	[76]	Milk HP	0.004	90.6	68.6	0.89
	[73]	Milk HP	0.056	100	100	–
	[76]	Serum HP	0.05	90.0	64.43	0.78
	[73]	Serum HP	0.03	90.0	100	–
Clinical mastitis	[115]	SAA	9.6	83.0	90.0	–
	[116]	SAA	26.41	79.2	95.0	0.95
	[115]	Serum HP	0.05	82.0	94.0	–
	[116]	Serum HP	0.09	86.8	80.0	0.93
	[115]	MAA	0.55	93.0	100	–
	[115]	Milk HP	0.02	86.0	100	-

^1^*Sen* Sensitivity (%), *Spec* specificity (%), *AUC* area under the curve

^2^*Alb* Albumin (g/dL), *SAA* Serum Amyloid A (μg/mL), *MAA* Milk Amyloid A (μg/mL), *HP* Haptoglobin (mg/mL)

Literature on APP levels in endometritis [88–90], urinary tract infections [83], hoof and leg disorders [91–93], foot and mouth disease [94, 95], paratuberculosis [96, 97], bovine viral disease [98], infections with *L. interrogans* [62], bovine besnoitiosis [71], *Babesia bigemina* [99], respiratory disease [100], subclinical and clinical ketosis [101], traumatic reticuloperitonitis [102], peritonitis [103] and calf diarrhoea [104] was found and summarized in Table 10. As seen in sows, SAA levels and HP levels increase after parturition, but stay elevated for a prolonged period in cows suffering from endometritis and metritis [90]. The exact period in which SAA and HP levels are significantly increased is equivocal since some discrepancy occurs in results and time period studied [88–90]. Regardless, it is concluded that SAA and HP could be reliable indicators of clinical endometritis and metritis [88–90], used to monitor treatment [89] and HP to evaluate reproductive performance [90]. Additionally, using a cut-off of 0.085 mg/mL for HP gave a sensitivity and specificity of 95% and 57.5%, respectively, between healthy cows and those with moderate endometritis [88]. For SAA a cut-off of 16.25 μg/mL gave a sensitivity and specificity of 95% and 55% between healthy cows and cows with moderate endometritis [88].

**Table 10:** Overview of acute phase protein (APP) levels in cows for other common diseases than mastitis

Reference	Age group^2^	Disease^3^	APP levels diseased cows^4^	APP levels healthy cows^4^
[90]	C	Metritis	4 weeks pp: SAA: 37^a^ HP: 0.21^a^ 2 months pp: SAA: 39^a^ HP: 0.23^a^	4 weeks pp: SAA: 26^b^ HP: 0.02^b^ 2 months pp: SAA: 27^b^ HP: 0.06^b^
[89]	C	Endometritis	40–90 days pp: SAA: 34.0–35.40^a^ HP: 0.08–0.082^a^	40–90 days pp: SAA: 16.8^b^ HP: 0.022^a^
[88]	C	Endometritis	28–32 days pp: SAA: 28.17^a^ HP: 0.183^a^	28–32 days pp: SAA: 14.24^b^ HP: 0.072^b^
[83]	C	UTI	SAA: 90.48^a^ HP: 4.32^a^ Fb: 1135^a^ AGP: 313.5^a^ Alb: 2.68^a^	SAA: 22.51^b^ HP: 0.12^b^ Fb: 354^b^ AGP: 237.0^b^ Alb: 2.71^a^
[93]	C	Interdigital dermatitis	Bacterial: SAA: 40.85^a^ HP: 0.46^a^ Non-bacterial: SAA: 23.62^a^ HP: 0.30^a^	SAA: 5.10^b^ HP: 0.09^b^
[92]	C	Lameness	CRP: 4.41^a^ SAA: 22.19^a^ HP: 0.22^a^ Fb: 397^a^	CRP: 0.61^b^ SAA: 8.89^b^ HP: 0.12^b^ Fb: 140^b^
[91]		Heel erosion (HE), acute laminitis (AL), sole ulcer (SU), digital dermatitis (DD), white line separation (WLS)	HE: SAA: 195.3^a^ HP: 0.29^a^ AL: SAA: 442.8^a^ HP: 0.59^a^ SU: SAA: 590.0^a^ HP: 0.59^a^ DD: SAA: 281.3^a^ HP: 0.37^a^ WLS: SAA: 95.6^a^ HP: 0.25^a^	HE: SAA: 111.35^a^ HP: 0.22^a^ AL: SAA: 111.35^b^ HP: 0.22^b^ SU: SAA: 111.35^b^ HP: 0.22^b^ DD: SAA: 111.35^b^ HP: 0.22^b^ WLS: SAA: 111.35^a^ HP: 0.22^a^
[105]	C	Digital dermatitis (DD), White line disease (WLD) and sole ulcers (SU)	DD: SAA: 28.8^a^ HP: 0.243^a^ WLD: SAA: 49.9^a^ HP: 0.239^a^ SU: SAA: 54.9^a^ HP: 0.277^a^	DD: SAA: 22.4^a^ HP: 0.148^a^ WLD: SAA: 22.4^a^ HP: 0.148^a^ SU: SAA: 22.4^b^ HP: 0.148^a^
[94]	C	FMD	SAA: 37.1^a^ HP: 0.41^a^ Fb: 464^a^	SAA: 4.5^b^ HP: 0.09^b^ Fb: 280^b^
[95]	C	FMD	SAA: 8.79^a^ HP: 0.507^a^	SAA: 6.86^b^ HP: 0.229^b^
[97]	C	Paratuberculosis	SAA: 12.54^a^ HP: 0.72^a^	SAA: 6.91^b^ HP: 0.33^b^
[96]	C	Paratuberculosis	SAA: 27.44^a^ HP: 0.238^a^ CRP: 45.45^b^	SAA: 15.69^a^ HP: 0.080^a^ CRP: 150.1^a^
[98]	C	BVD	SAA: 49.98^a^ HP: 0.357^a^	SAA: 13.64^b^ HP: 0.176^b^
[101]	C	Subclinical (SCK) and clinical ketosis (CK)	SCK: SAA: NA^a^ HP: NA^a^ CK: SAA: NA^a^ HP: NA^a^	SAA:NA^b^ HP: NA^b^
[102]	C	Traumatic reticuloperitonitis	SAA:179.45^a^ HP: 1.31^a^ Fb: 518.86^a^	SAA: 4.49^b^ HP: 0.08^b^ Fb: 206^b^
[103]	C	Peritonitis	HP: 1.88^a^ Fb: 379.7^a^ Alb: 3.6^a^	HP: 0.03^b^ Fb: 293.7^b^ Alb: 3.51^a^
[62]	C	*L. interrogans*	SAA: 32.38^a^ HP: 3.77^a^ Alb: 0.291^a^	SAA: 20.1^b^ HP: 1.37^b^ Alb: 0.297^a^
[71]	C	Bovine besnoitiosis	HP: NA^a^ Alb: NA^b^	HP: NA^a^ Alb: NA^a^
[99]	C	Babesia bigemina	SAA: 1.834^a^ HP: 0.41^a^ Fb: 820.6^b^	SAA: 0.0479^b^ HP: 0.15^b^ Fb: 639.15^a^
[117]	C	Lumpy skin disease	SAA:1.27^a^ HP: 0.100^a^ Alb: 3.08^b^	SAA: 0.85^b^ HP: 0.079^b^ Alb: 3.6^a^
[100]	C	Respiratory disease	Upper respiratory/ lower respiratory: HP: 0.50/0.48^a^ Fb: 922/976^b^	HP: 0.34^b^ Fb: 580^a^
[112]	Ca	SIRS	HP: 0.29^a^	HP: 0.22^a^
[104]	Ca	Diarrhoea	SAA: bacterial: 2.77^b^ viral: 3.57^bc^	SAA: 0.95^a^

^1^means—columns with different superscripts differ significantly (P < 0.05)

^2^*Ca* Calves, *C* Cow

^3^*UTI* Urinary tract infections, *FMD* Foot and mouth disease, *BVD* Bovine viral disease, *SIRS* systemic inflammatory response syndrome

^4^*CRP* C-reactive protein (μg/mL), *SAA* Serum Amyloid A (μg/mL), *MAA* Milk Amyloid A (μg/mL), *HP* Haptoglobin (mg/mL), *Fb* Fibrinogen (mg/dL), *Alb* Albumin (g/dL), *AGP* Alpha 1-acid glycoprotein (μg/mL)

One or two studies per disease were found for urinary tract infections (UTIs), foot and mouth disease (FMD), traumatic reticuloperitonitis and bovine viral disease (BVD) and the studies suggest a significant increase in HP and SAA for all four diseases [83, 94, 95, 98, 102], a significant increase in fibrinogen in traumatic reticuloperitonitis, FMD and UTIs [83, 94, 102] and a significant increase in AGP in UTIs [83]. Furthermore, a study suggests a high diagnostic accuracy of Fb, AGP, HP and SAA in UTIs, with AUC being between 0.93 and 0.98 for all APPs and a high prognostic accuracy of SAA and HP (AUC > 0.95) [83]. The association being that a higher HP or SAA level corresponded to poor treatment response after treatment with antibiotics.

Generally speaking, lameness and claw disorders lead to significant increases in HP, SAA [91–93], Fb and CRP [92] compared to healthy controls. However, when studying the aetiology behind claw lesions, heel erosions and white line separation seem to provide a lower and non-significant increase in HP and SAA compared to acute laminitis, sole ulcers and digital dermatitis [91]. A finding partly supported by an Estonian study which found that sole ulcers led to increased SAA levels but not HP levels and that digital dermatitis had no significant effect on HP or SAA level [105]. Additionally, when looking at interdigital laminitis, lameness associated with *F. necrophorum* provided a quantitatively higher level of HP and SAA compared to lameness not associated with *F. necrophorum.* Due to low sample size in the *F. necrophorum* positive group (n = 4) a statistical comparison was not computed [93].

Paratuberculosis in cattle is a non-curable enteric disease caused by *M. avium* spp. *paratuberculosis* (MAP)*.* The disease is associated with chronic diarrhoea, and compared to healthy controls, MAP-seropositive animals with chronic diarrhoea had significant alterations in albumin levels but not in HP and SAA [96]. The lack of a response in SAA and HP could be due to lower total protein levels in serum caused by the protein-losing enteropathy associated with paratuberculosis [106, 107]. However, when looking at different pathological forms of paratuberculosis in cattle, multifocal and diffuse paucibacillary lesions but not focal and diffuse multibacillary lesions, lead to significantly increased SAA and Hp levels [97]. This suggests a difference in APP pattern between lesion types. Another explanation to a lack of response in HP and SAA in paratuberculosis could be the chronic nature of the disease, since chronic disease has been shown to relate to lower APP levels compared to acute disease in cattle [108].

Investigations on the relationship between APP levels and health status in calves discovered that inflammatory disorders such as respiratory and gastrointestinal disease was connected to elevated APP responses [104, 109–113], and fibrinogen and HP levels mirrored the faecal score with Fb and HP increasing with increased looseness of the faeces, a pattern not seen in SAA [112]. Furthermore, *E. coli* diarrhoea gave a higher SAA response than coronavirus based diarrhoea [104]. The effect of sepsis on acute phase proteins was reviewed recently, and it concludes that acute phase proteins are unspecific for sepsis in both pigs, horses and cows, but can provide information on disease development and prognosis [25]. The review includes a study on eight calves with significant HP increases in calves with experimentally induced endotoxemia [25], whereas a study on naturally occurring systemic inflammatory response syndrome in 102 calves showed no increase in HP compared to non-SIRS calves [113].

#### Summary—cattle

The major acute phase proteins in cattle are serum or milk amyloid A and HP, and the majority of literature reports oncases of clinical and subclinical mastitis. Fb is, as for horses, commonly studied and is significantly lower in diseases such as subclinical and clinical mastitis, urinary tract infections and lameness, which could be valuable for the purposes of diagnostics and treatment. Mastitis, both experimentally induced and field cases, resulted in significant changes in serum and milk amyloid A and HP, and Gram-negative bacteria appeared to produce a higher APP response than Gram-positive bacteria, with few exceptions. Significant increases in serum and milk amyloid A and HP are seen in subclinical mastitis, and the level corresponds to the number of udder quarters affected. Using serum and milk HP and amyloid A as a diagnostic marker of subclinical mastitis yielded a sensitivity of more than 90% and a specificity ranging from 56.66 to 100% depending on the cut-off used. Furthermore, one study suggested that SAA could be used to differentiate between subclinical cases with and without positive bacteriology, and HP could be used to differentiate *S. aureus* cases that will self-resolve. For some other diseases than mastitis, HP and SAA are elevated, except for besnoitiosis, some claw lesions and paratuberculosis where evidence is equivocal. Furthermore, studies suggested that SAA and HP could be used to monitor and predict treatment response in uterine tract infections and endometritis and that lameness associated with *F. necrophorum* infections causes higher HP and SAA responses than non-*F. necrophorum* interdigital laminitis. In calves, diarrhoea of bacterial origin yielded a higher SAA response than viral diarrhoea.

## Conclusions

Much research has been put into identifying which APPs are elevated or decreased in diseases affecting, pigs, horses and cows and correlating clinical signs with the APR. Generally, most diseases and clinical signs of disease initiate an APR in the moderate to major APPs for all species. Few studies, however, evaluated the diagnostic and prognostic potential. Research on the APR in humans has shown that APPs can be used diagnostically e.g., to comprehend the seriousness of a condition, to distinguish infectious from non-infectious causes of disease, or bacterial from viral causes or guide antibiotic treatment initiation and efficacy. This review found limited evidence within pigs, horses, and cattle on either of these subjects.

Eleven studies used ROC-curve analysis to establish the diagnostic performance of an APP in differentiating diseased from non-diseased animals. Six of the eleven studies targeted clinical or subclinical mastitis. Using APP levels to distinguish between healthy and sick animals is valuable in conditions where welfare or production outcomes are affected before clinical signs arise as is the case with subclinical mastitis. However, the value is limited in diseases such as lumpy skin disease where the diagnosis can be determined from clinical signs alone or in multifactorial disease where both bacterial, viral and non-infectious causes lead to similar clinical signs e.g., diarrhoea or lameness. In the latter cases quantification of the acute phase level is valuable for the veterinarian or the farmer if it can be correlated to seriousness of the disease, prognosis, aetiology, or treatment.

This review identified 10 studies relating acute phase protein levels to aetiology, three to antibiotic treatment efficacy, one to prognosis and two to seriousness of disease. However, these studies concern different species and diseases, hence it is difficult to definitively infer upon the usefulness of APPs to mitigate unnecessary antibiotic use. Nevertheless, with the limited amount of evidence in mind, CRP might have the potential to differentiate between bacterial and non-bacterial causes of disease in pigs. SAA could reflect underlying aetiology in horses, and in mastitis and neonatal diarrhoea in cattle.

Since reducing antibiotic usage is of high priority, tools to guide initiation and termination would be of great interest. Identification of APPs could aid in separating bacterial from viral or non-infectious causes of disease, predict prognosis and be used to evaluate antibiotic treatment efficacy. More evidence should be collected for each disease within a species where mitigation of antibiotic usage is relevant and underlying aetiology cannot be determined by the clinical signs alone or where pre-treatment diagnostics are not viable. It is the authors’ belief that to determine the viability of using APPs to mitigate unnecessary antibiotic use, more efforts should be put into correlating APP levels and underlying aetiology, establishing cut-off values for infectious and non-infectious cases, evaluate the diagnostic performance of APPs and evaluate any adverse effects of using APPs to determine treatment strategy.

## Data Availability

Not applicable.

## References

[1] Yaeger MJ, Van Alstein WG. Respiratory system. In: Zimmerman JJ, Karriker LA, Ramirez A, Schwartz KJ, Stevenson GW, Zhang J, editors. Diseases of swine. 11th ed. Hoboken, NJ, USA: Wiley Blackwell; 2019. p. 393–407.

[2] Ruegg PL. A 100-year review: mastitis detection, management, and prevention. J Dairy Sci. 2017;100:10381–97. 29153171 10.3168/jds.2017-13023

[3] Ramirez A. Differential diagnosis of diseases. In: Zimmerman JJ, Karriker LA, Ramirez A, Schwartz KJ, Stevenson GW, Zhang J, editors. Diseases of swine. 11th ed. Hoboken, NJ, USA: Wiley Blackwell; 2019. p. 59–74.

[4] Madson DM, Arruda PHE, Arruda BL. Nervous and Locomotor system. In: Zimmerman JJ, Karriker LA, Ramirez A, Schwartz KJ, Stevenson GW, Zhang J, editors. Diseases of swine. 11th ed. Hoboken, NJ, USA: Wiley Blackwell; 2019. p. 339–72.

[5] Wilm J, Svennesen L, Eriksen EØ, Halasa T, Krömker V. Veterinary treatment approach and antibiotic usage for clinical mastitis in Danish dairy herds. Antibiotics. 2021. 10.3390/antibiotics10020189. 33671911 10.3390/antibiotics10020189 PMC7918953

[6] Servia-Dopazo M, Taracido-Trunk M, Figueiras A. Non-clinical factors determining the prescription of antibiotics by veterinarians: a systematic review. Antibiotics. 2021. 10.3390/antibiotics10020133. 33573109 10.3390/antibiotics10020133 PMC7912449

[7] World Health Organization. Global action plan on antimicrobial resistance. Geneva: Scwitzerland; 2015. 10.7196/samj.9644 26242647

[8] World Organisation for Animal Health. The OIE strategy on antimicrobial resistance and the prudent use of antimicrobials. 2016

[9] Castell JV, Gomez-Lechon MJ, David M, Andus T, Geiger T, Trullenque R, et al. Interleukin-6 is the major regulator of acute phase protein synthesis in adult human hepatocytes. Fed Eur Biomed Sci. 1989;242:237–9. 10.1016/0014-5793(89)80476-4 2464504

[10] Gruys E, Toussaint MJM, Niewold TA, Koopmans SJ. Acute phase reaction and acute phase proteins. J Zhejiang Univ Sci. 2005;6B:1045–56. 10.1631/jzus.2005.B1045 PMC1390650 16252337

[11] Eckersall PD, Bell R. Acute phase proteins: Biomarkers of infection and inflammation in veterinary medicine. Vet J. 2010;185:23–7. 20621712 10.1016/j.tvjl.2010.04.009

[12] Cui Y, Wang C, Hao Y, Gu X, Wang H. Chronic heat stress induces acute phase responses and serum metabolome changes in finishing pigs. Animals. 2019. 10.3390/ani9070395. 31261803 10.3390/ani9070395 PMC6680871

[13] Kaiser M, Jacobsen S, Andersen PH, Bækbo P, Cerón JJ, Dahl J, et al. Hormonal and metabolic indicators before and after farrowing in sows affected with postpartum dysgalactia syndrome. BMC Vet Res. 2018. 10.1186/s12917-018-1649-z. 30404636 10.1186/s12917-018-1649-z PMC6223068

[14] Wierzchosławski K, Kwit K, Pejsak Z, Pomorska-Mól M. Selected serum acute-phase proteins in peripartum sows and evaluation of their diagnostic usefulness. Anim Reprod Sci. 2018;191:44–55. 29433895 10.1016/j.anireprosci.2018.02.003

[15] Arfuso F, Giannetto C, Fazio F, Panzera F, Piccione G. Training program intensity induces an acute phase response in clinically healthy horses. J Equine Vet Sci. 2020. 10.1016/j.jevs.2020.102986. 32303313 10.1016/j.jevs.2020.102986

[16] Cywinska A, Cywinska C, Szarska E, Górecka R, Witkowski L, Hecold M, et al. Acute phase protein concentrations after limited distance and long distance endurance rides in horses. Res Vet Sci. 2012;93:1402–6. 22390917 10.1016/j.rvsc.2012.02.008

[17] Fontela PS, O’Donnell S, Papenburg J. Can biomarkers improve the rational use of antibiotics? Curr Opin Infect Dis. 2018. 10.1097/QCO.0000000000000467. 29794541 10.1097/QCO.0000000000000467

[18] Simon L, Gauvin F, Amre DK, Saint-Louis P, Lacroix J. Serum procalcitonin and C-Reactive protein levels as markers of bacterial infection: a systematic review and meta-analysis. Clin Infect Dis. 2014;39:206–17. 10.1086/421997 15307030

[19] Do NTT, Ta NTD, Tran NTH, Than HM, Vu BTN, Hoang LB, et al. Point-of-care C-reactive protein testing to reduce inappropriate use of antibiotics for non-severe acute respiratory infections in Vietnamese primary health care: a randomised controlled trial. Lancet Glob Heal. 2016;4:633–41. 10.1016/S2214-109X(16)30142-5 PMC4985565 27495137

[20] Downes KJ, Weiss SL, Gerber JS, Klieger SB, Fitzgerald JC, Balamuth F, et al. A pragmatic biomarker-driven algorithm to guide antibiotic use in the pediatric intensive care unit: the optimizing antibiotic strategies in sepsis (OASIS) study. J Pediatric Infect Dis Soc. 2017;6:134–41. 27147715 10.1093/jpids/piw023 PMC5907860

[21] Smedemark SA, Llor C, Aabenhus R, Fournaise A, Jørgensen KJ, Olsen O. Biomarkers as point-of-care tests to guide prescription of antibiotics in people with acute respiratory infections in primary care. Cochrane Database Syst Rev. 2022. 10.1002/14651858.CD010130.pub3. 36250577 10.1002/14651858.CD010130.pub3 PMC9575154

[22] Ritchie B, Porritt K, Marin T, Williams N. Diagnostic test accuracy of serum procalcitonin compared with C-reactive protein for bone and joint infection in children and adolescents: a systematic review and meta-analysis. JBI Evid Synth. 2021;19:3209–37. 34402489 10.11124/JBIES-20-00357

[23] Yo CH, Hsieh PS, Lee SH, Wu JY, Chang SS, Tasi KC, et al. Comparison of the test characteristics of procalcitonin to C-Reactive protein and leukocytosis for the detection of serious bacterial infections in children presenting with fever without source: a systematic review and meta-analysis. Ann Emerg Med. 2012;60:591–600. 22921165 10.1016/j.annemergmed.2012.05.027

[24] Van den Bruel A, Thompson MJ, Haj-Hassan T, Stevens R, Moll H, Lakhanpaul M, et al. Diagnostic value of laboratory tests in identifying serious infections in febrile children: systematic review. BMJ. 2011. 10.1136/bmj.d3082. 21653621 10.1136/bmj.d3082

[25] López-Martínez MJ, Franco-Martínez L, Martínez-Subiela S, Cerón JJ. Biomarkers of sepsis in pigs, horses and cattle: from acute phase proteins to procalcitonin. Anim Heal Res Rev. 2022;23:82–99. 10.1017/S1466252322000019 35795920

[26] Sorensen NS, Tegtmeier C, Andresen LO, Piñeiro M, Toussaint MJM, Campbell FM, et al. The porcine acute phase protein response to acute clinical and subclinical experimental infection with *Streptococcus**suis*. Vet Immunol Immunopathol. 2006;113:157–68. 16774789 10.1016/j.vetimm.2006.04.008

[27] Asai T, Mori M, Okada M, Uruno K, Yazawa S, Shibata I. Elevated serum haptoglobin in pigs infected with porcine reproductive and respiratory syndrome virus. Vet Immunol Immunopathol. 1999;70:143–8. 10507295 10.1016/S0165-2427(99)00069-0

[28] Gómez-Laguna J, Gutiérrez A, Pallarés FJ, Salguero FJ, Cerón JJ, Carrasco L. Haptoglobin and C-reactive protein as biomarkers in the serum, saliva and meat juice of pigs experimentally infected with porcine reproductive and respiratory syndrome virus. Vet J. 2010;185:83–7. 20554227 10.1016/j.tvjl.2010.04.018

[29] Hultén C, Johansson E, Fossum C, Wallgren P. Interleukin 6, serum amyloid a and haptoglobin as markers of treatment efficacy in pigs experimentally infected with *Actinobacillus**pleuropneumoniae*. Vet Microbiol. 2003;95:75–89. 12860078 10.1016/S0378-1135(03)00136-6

[30] Pomorska-Mól M, Markowska-Daniel I, Kwit K, Stępniewska K, Pejsak Z. Profile of the porcine acute-phase proteins response following experimental co-infection with H3N2 swine influenza virus and *Pasteurella**multocida*. Biomarkers. 2015;20:189–95. 26161700 10.3109/1354750X.2015.1061600

[31] Pomorska-Mól M, Markowska-Daniel I, Kwit K, Stepniewska K, Pejsak Z. C-reactive protein, haptoglobin, serum amyloid A and pig major acute phase protein response in pigs simultaneously infected with H1N1 swine influenza virus and *Pasteurella multocida*. BMC Vet Res. 2013;9:1–9. 23332090 10.1186/1746-6148-9-14 PMC3554491

[32] Pomorska-Mól M, Urbaniak K, Markowska-Daniel I. Porcine acute phase protein response to experimental infection with *Bordetella**bronchiseptica*. Bull Vet Inst Pulw. 2011;55:371–5.

[33] Pomorska-Mól M, Markowska-Daniel I, Kwit K, Stepniewska K, Pejsak Z. Kinetics of the response of four positive acute phase proteins in pigs experimentally infected with toxigenic *Pasteurella**multocida*. Vet Microbiol. 2011;152:429–35. 21676559 10.1016/j.vetmic.2011.05.032

[34] Pomorska-Mól M, Markowska-Daniel I, Kwit K, Urbaniak K, Pejsak Z. Correlation between serum acute phase proteins, lung pathology, and disease severity in pigs experimentally co-infected with H3N2 swine influenza virus and *bordetella**bronchiseptica*. Bull Vet Inst Pulawy. 2015;59:1–7. 10.1515/bvip-2015-0001

[35] Pomorska-Mol M, Markowska-Daniel I, Kwit K. Acute phase protein response in pigs experimentally co-infected with swine influenza virus and *Bordetella**bronchiseptica*. Cent J Immunol. 2012;37:221–6. 10.5114/ceji.2012.30818

[36] Pomorska-Mól M, Dors A, Kwit K, Kowalczyk A, Stasiak E, Pejsak Z. Kinetics of single and dual infection of pigs with swine influenza virus and *Actinobacillus**pleuropneumoniae*. Vet Microbiol. 2017;201:113–20. 28284596 10.1016/j.vetmic.2017.01.011

[37] Heegaard PMH, Klausen J, Nielsen JP, González-Ramón N, Piñeiro M, Lampreave F, et al. The porcine acute phase response to infection with *Actinobacillus**pleuropneumoniae*. Haptoglobin, C-Reactive protein, major acute phase protein and serum amyloid A protein are sensitive indicators of infection. Comp Biochem Physiol. 1998;119:365–73. 10.1016/S0305-0491(97)00362-3 9629669

[38] Skovgaard K, Mortensen S, Boye M, Poulsen KT, Campbell FM, Eckersall PD, et al. Rapid and widely disseminated acute phase protein response after experimental bacterial infection of pigs. Vet Res. 2009. 10.1051/vetres/2009006. 19236838 10.1051/vetres/2009006 PMC2695040

[39] Lauritzen B, Lykkesfeldt J, Skaanild MT, Angen Ø, Nielsen JP, Friis C. Putative biomarkers for evaluating antibiotic treatment: an experimental model of porcine *Actinobacillus**pleuropneumoniae* infection. Res Vet Sci. 2003;74:261–70. 12726745 10.1016/S0034-5288(03)00028-6

[40] Knura-Deszczka S, Lipperheide C, Petersen B, Jobert JL, Berthelot-Hérault F, Kobisch M, et al. Plasma haptoglobin concentration in swine after challenge with *Streptococcus**suis*. J Vet Med. 2002;49:240–4. 10.1046/j.1439-0450.2002.00556.x 12121045

[41] Lüthje FL, Blirup-Plum SA, Møller NS, Heegaard PMH, Jensen HE, Kirketerp-Møller K, et al. The host response to bacterial bone infection involves a local upregulation of several acute phase proteins. Immunobiology. 2020. 10.1016/j.imbio.2020.151914. 32098686 10.1016/j.imbio.2020.151914

[42] Carpintero R, Piñeiro M, Andrés M, Iturralde M, Alava MA, Heegaard PMH, et al. The concentration of apolipoprotein A-I decreases during experimentally induced acute-phase processes in pigs. Infect Immun. 2005;73:3184–7. 15845530 10.1128/IAI.73.5.3184-3187.2005 PMC1087351

[43] Zhu Y, Österlundh I, Hultén F, Magnusson U. Tumor necrosis factor-α, interleukin-6, serum amyloid a, haptoglobin, and cortisol concentrations in sows following intramammary inoculation of *Escherichia**coli*. Am J Vet Res. 2004;65:1434–9. 15524332 10.2460/ajvr.2004.65.1434

[44] Belser JA, Gustin KM, Katz JM, Maines TR, Tumpey TM. Comparison of traditional intranasal and aerosol inhalation inoculation of mice with influenza a viruses. Virology. 2015;481:107–12. 25771498 10.1016/j.virol.2015.02.041 PMC5725743

[45] Guan J, Fu Q, Sharif S. Replication of an H9N2 avian influenza virus and cytokine gene expression in chickens exposed by aerosol or intranasal routes. Avian Dis. 2015;59:263–8. 26473677 10.1637/10972-110714-Reg

[46] Jacobson M, Lindberg JE, Lindberg R, Af Segerstad CH, Wallgren P, Fellström C, et al. Intestinal cannulation: model for study of the midgut of the pig. Comp Med. 2001;51:163–70. 11922181

[47] Petersen HH, Ersbøll AK, Jensen CS, Nielsen JP. Serum-haptoglobin concentration in Danish slaughter pigs of different health status. Prev Vet Med. 2002;54:325–35. 12163249 10.1016/S0167-5877(02)00054-5

[48] SPF-SUND. SPF-diseases. https://spfsus.dk/en. Accessed 29 Nov 2023.

[49] Parra MD, Fuentes P, Tecles F, Martínez-Subiela S, Martínez JS, Muñoz A, et al. Porcine acute phase protein concentrations in different diseases in field conditions. J Vet Med. 2006;53:488–93. 10.1111/j.1439-0450.2006.01002.x 17123428

[50] Hu L, Shi Q, Shi M, Liu R, Wang C. Diagnostic value of PCT and CRP for detecting serious bacterial infections in patients with fever of unknown origin: a systematic review and meta-analysis. Appl Immunohistochem Mol Morphol. 2017;25:61–9. 10.1097/PAI.0000000000000552 28885233

[51] Petersen HH, Dideriksen D, Christiansen BM, Nielsen JP. Serum haptoglobin concentration as a marker of clinical signs in finishing pigs. Vet Rec. 2002;151:85–9. 12164226 10.1136/vr.151.3.85

[52] Loewenstein F, Becker S, Kuehling J, Schrade H, Lechner M, Ringseis R, et al. Inflammation and necrosis syndrome is associated with alterations in blood and metabolism in pigs. BMC Vet Res. 2022. 10.1186/s12917-021-03107-1. 35045844 10.1186/s12917-021-03107-1 PMC8767723

[53] Kemper N. Update on postpartum dysgalactia syndrome in sows. J Anim Sci. 2020;98:117–25. 10.1093/jas/skaa135 PMC7433910 32810252

[54] Oravainen J, Heinonen M, Seppä-Lassila L, Orro T, Tast A, Virolainen JV, et al. Factors affecting fertility in loosely housed sows and gilts: vulvar discharge syndrome, environment and acute-phase proteins. Reprod Domest Anim. 2006;41:549–54. 17107516 10.1111/j.1439-0531.2006.00713.x

[55] Mirko CP, Bilkei G. Acute phase proteins, serum cortisol and preweaning litter performance in sows suffering from periparturient disease. Acta Vet. 2004;54:153–61. 10.2298/AVB0403153M

[56] Canisso IF, Ball BA, Cray C, Williams NM, Scoggin KE, Davolli GM, et al. Serum amyloid A and haptoglobin concentrations are increased in plasma of mares with ascending placentitis in the absence of changes in peripheral leukocyte counts or fibrinogen concentration. Am J Reprod Immunol. 2014;72:376–85. 24916762 10.1111/aji.12278

[57] Hultén C, Grönlund U, Hirvonen J, Tulamo RM, Suominen MM, Marhaug G, et al. Dynamics in serum of the inflammatory markers serum amyloid A (SAA), haptoglobin, fibrinogen and α2-globulins during induced noninfectious arthritis in the horse. Equine Vet J. 2002;34:699–704. 12455841 10.2746/042516402776250405

[58] Garcia Motta R, de Souza Araújo Martins L, Costa Da Silva R, Vinícius Ramos Portilho F, Guerra Trevizan S, da Rocha MA, et al. Etiology, multidrug resistance, and acute-phase proteins biomarkers as in equine septic arthritis. Ciência Rural. 2020. 10.1590/0103-8478cr20200386. 10.1590/0103-8478cr20200386

[59] Hultén C, Demmers S. Serum amyloid A (SAA) as an aid in the management of infectious disease in the foal: comparison with total leucocyte count, neutrophil count and fibrinogen. Equine Vet J. 2002;34:693–8. 12455840 10.2746/042516402776250360

[60] Bullone M, de Lagarde M, Vargas A, Lavoie JP. Serum surfactant protein D and haptoglobin as potential biomarkers for inflammatory airway disease in horses. J Vet Intern Med. 2015;29:1707–11. 26289543 10.1111/jvim.13602 PMC4895656

[61] Pusterla N, Barnum S, Hall JA, Marshall-Lund L, Gebhart C. Investigation of the usefulness of serum amyloid A in supporting the diagnosis of equine proliferative enteropathy. J Equine Vet Sci. 2020. 10.1016/j.jevs.2020.103151. 32797779 10.1016/j.jevs.2020.103151

[62] Niroomandi E, Maleki S, Abdollahpour G, Zakian A, Ahmadvand H. The effect of natural infection with different *Leptospira**interrogans* serovars on oxidative stress biomarkers and acute-phase responses in horses and cattle. Vet Clin Pathol. 2022;51:84–92. 35179227 10.1111/vcp.13042

[63] El-Deeb W, Iacob O, Fayez M, Elgioushy M, Shawaf T, Ibrahim A. Acute phase proteins, interleukin-6, tumor necrosis factor, nitric oxide and oxidative stress markers in horses with cutaneous habronemosis under field condition. Vet Parasitol. 2018;255:20–5. 29773131 10.1016/j.vetpar.2018.03.023

[64] Rodríguez R, Cerón JJ, Riber C, Castejón F, Gómez-Díez M, Serrano-Rodríguez JM, et al. Acute phase proteins in Andalusian horses infected with *Theileria**equi*. Vet J. 2014;202:182–3. 25086769 10.1016/j.tvjl.2014.07.003

[65] Mittelman NS, Stefanovski D, Johnson AL. Utility of C-reactive protein and serum amyloid a in the diagnosis of equine protozoal myeloencephalitis. J Vet Intern Med. 2018;32:1726–30. 30216559 10.1111/jvim.15254 PMC6189384

[66] Jacobsen S, Thomsen MH, Nanni S. Concentrations of serum amyloid a in serum and synovial fluid from healthy horses and horses with joint disease. Am J Vet Res. 2006;67:1738–42. 17014325 10.2460/ajvr.67.10.1738

[67] Blum SE, Heller DE, Jacoby S, Krifuks O, Merin U, Silanikove N, et al. Physiological response of mammary glands to *Escherichia**coli* infection: a conflict between glucose need for milk production and immune response. Sci Rep. 2020. 10.1038/s41598-020-66612-7. 32541828 10.1038/s41598-020-66612-7 PMC7296043

[68] Bannerman DD, Kauf ACW, Paape MJ, Springer HR, Goff JP. Comparison of Holstein and Jersey innate immune responses to *Escherichia**coli* intramammary infection. J Dairy Sci. 2008;91:2225–35. 18487645 10.3168/jds.2008-1013

[69] Grönlund U, Sandgren CH, Waller KP. Haptoglobin and serum amyloid a in milk from dairy cows with chronic sub-clinical mastitis. Vet Res. 2005;36:191–8. 15720972 10.1051/vetres:2004063

[70] Hirvonen J, Eklund K, Teppo AM, Huszenicza G, Kulcsar M, Saloniemi H, et al. Acute phase response in dairy cows with experimentally induced *Escherichia**coli* mastitis. Acta Vet Scand. 1999;40:35–46. 10418194 10.1186/BF03547039 PMC8043231

[71] González-Barrio D, Huertas-López A, Diezma-Díaz C, Ferre I, Cerón JJ, Ortega-Mora LM, et al. Changes in serum biomarkers of inflammation in bovine besnoitiosis. Parasit d Vectors. 2021. 10.1186/s13071-021-04991-0. 10.1186/s13071-021-04991-0 PMC8459460 34551803

[72] Cray C, Zaias J, Altman NH. Acute phase response in animals: a review. Comp Med. 2009;59:517–26. 20034426 PMC2798837

[73] Kumar P, Sharma A, Chhabra R, Sharma R, Sindhu N, Deora A. A comparative study on acute phase proteins and other diagnostic tests for detection of subclinical mastitis in cows. Vet Pract. 2015;16.

[74] Sahu S, Maiti SK, Raghuvanshi PDS. Effect of subclinical mastitis on fibrinogen, orotic acid and some enzymes in the milk of dairy cows. Ind J Vet Sci Biotech. 2020;16:17–20.

[75] Kovacevic MF, Stevanovic J, Stevanov MP, Debeljak JM, Knezevic M, Mijacevic Z, et al. Acute phase protein response in cows with *staphylococcus**aureus* subclinical mastitis. Acta Vet. 2010;60:205–16. 10.2298/AVB1003205K

[76] Safi S, Khoshvaghti A, Jafarzadeh SR, Bolourchi M, Nowrouzian I. Acute phase proteins in the diagnosis of bovine subclinical mastitis. Vet Clin Pathol. 2009;38:471–6. 19548974 10.1111/j.1939-165X.2009.00156.x

[77] Shirazi-Beheshtiha SH, Safi S, Rabbani V, Bolourchi M, Ameri M, Khansari MR. The diagnostic value of determination of positive and negative acute phase proteins in milk from dairy cows with subclinical mastitis. Comp Clin Path. 2012;21:999–1003. 10.1007/s00580-011-1216-5

[78] Sadek K, Saleh E, Ayoub M. Selective, reliable blood and milk bio-markers for diagnosing clinical and subclinical bovine mastitis. Trop Anim Health Prod. 2017;49:431–7. 27915437 10.1007/s11250-016-1190-7

[79] Bochniarz M, Zdzisinska B, Wawron W, Szczubial M, Dabrowaki R. Milk and serum IL-4, IL-6, IL-10, and amyloid a concentrations in cows with subclinical mastitis caused by coagulase-negative staphylococci. J Dairy Sci. 2017;100:9674–80. 28964518 10.3168/jds.2017-13552

[80] Nazifi S, Haghkhah M, Asadi Z, Ansari-Lari M, Tabandeh MR, Esmailnezhad Z, et al. Evaluation of sialic acid and acute phase proteins (haptoglobin and serum amyloid A) in clinical and subclinical bovine mastitis. Pak Vet J. 2011;31:55–9.

[81] Tabatabaee N, Heidarpour M, Khoramian B. Milk metabolites, proteins and oxidative stress markers in dairy cows suffering from *Staphylococcus**aureus* subclinical mastitis with or without spontaneous cure. J Dairy Res. 2021;88:326–9. 34382922 10.1017/S0022029921000613

[82] Chouhan D, Aich R, Jain RK, Chhabra D. Acute phase protein as biomarker for diagnosis of sub-clinical mastitis in cross-bred cows. Indian J Anim Res. 2021;55:193–8.

[83] El-Deeb WM, Elmoslemany AM. Acute phase proteins as biomarkers of urinary tract infection in dairy cows: diagnostic and prognostic accuracy. Jpn J Vet Res. 2016;64:57–66. 27348889

[84] Kumar P, Sharma A, Chhabra R, Sharma R, Sindhu N, Anupama. Observations on acute phase proteins and various diagnostic tests for udder health. Haryana Vet. 2012;51:89–91.

[85] Wenz JR, Fox LK, Muller FJ, Rinaldi M, Zeng R, Bannerman DD. Factors associated with concentrations of select cytokine and acute phase proteins in dairy cows with naturally occurring clinical mastitis. J Dairy Sci. 2010;93:2458–70. 20494154 10.3168/jds.2009-2819

[86] Dalanezi FM, Schmidt EMS, Joaquim SF, Guimarães FF, Guerra ST, Lopes BC, et al. Concentrations of acute-phase proteins in milk from cows with clinical mastitis caused by different pathogens. Pathogens. 2020. 10.3390/pathogens9090706. 32867136 10.3390/pathogens9090706 PMC7559481

[87] Pyörälä S, Hovinen M, Simojoki H, Fitzpatrick J, Eckersall PD, Orro T. Acute phase proteins in milk in naturally acquired bovine mastitis caused by different pathogens. Vet Rec. 2011. 10.1136/vr.d1120. 21558129 10.1136/vr.d1120

[88] Kaya S, Merhan O, Kacar C, Colak A, Bozukluhan K. Determination of ceruloplasmin, some other acute phase proteins, and biochemical parameters in cows with endometritis. Vet world. 2016;9:1056–62. 27847413 10.14202/vetworld.2016.1056-1062 PMC5104712

[89] Biswal SS, Das S, Balasubramanian S, Mohanty DN, Sethy K, Dasgupta M. Serum amyloid a and haptoglobin levels in crossbred cows with endometritis following different therapy. Vet World. 2014;7:1066–70. 10.14202/vetworld.2014.1066-1070

[90] Chan JPW, Chang CC, Hsu WL, Liu WB, Chen TH. Association of increased serum acute-phase protein concentrations with reproductive performance in dairy cows with postpartum metritis. Vet Clin Pathol. 2010;39:72–8. 19747184 10.1111/j.1939-165X.2009.00182.x

[91] Ilievska K, Atanasov B, Dovenski T, Smolac O, Stojanov B, Trojachanec P. Acute phase proteins—as indicators of claw diseases in dairy cattle. Maced Vet Rev. 2019;42:95–100. 10.2478/macvetrev-2019-0011

[92] Bagga A, Randhawa SS, Sharma S, Bansal BK. Acute phase response in lame crossbred dairy cattle. Vet World. 2016;9:1204–8. 27956769 10.14202/vetworld.2016.1204-1208 PMC5146298

[93] Nazifi S, Esmailnezhad Z, Haghkhah M, Ghadirian S, Mirzaei A. Acute phase response in lame cattle with interdigital dermatitis. World J Microbiol Biotechnol. 2012;28:1791–6. 22805961 10.1007/s11274-011-0995-9

[94] Nazifi S, Ansari-Lari M, Ghafari N, Mohtarami S, Ghezelbash A, Tabandeh MR. Evaluation of sialic acids, TNF-α, INF-γ, and acute-phase proteins in cattle infected with foot-and-mouth disease. Comp Clin Path. 2012;21:23–8. 10.1007/s00580-010-1059-5

[95] Mallick S, Subramaniam S, Biswal JK, Ranjan R, Mohapatra JK, Sahoo AP. Short communication: preliminary observations on the serum levels of HSP70 and its correlation with serum cortisol, thyroid hormones, and acute-phase protein concentration in cattle naturally infected with foot-and-mouth disease virus. Trop Anim Health Prod. 2021. 10.1007/s11250-021-02814-z. 34292411 10.1007/s11250-021-02814-z

[96] Nagy O, Tóthová C, Mudroň P. The impact of chronic diarrhoea in *Mycobacterium**avium* subsp. *paratuberculosis* seropositive dairy cows on serum protein fractions and selected acute phase proteins. J Appl Anim Res. 2020;48:14–20. 10.1080/09712119.2020.1714631

[97] Espinosa J, de la Morena R, Benavides J, García-Pariente C, Fernández M, Tesouro M, et al. Assessment of acute-phase protein response associated with the different pathological forms of bovine paratuberculosis. Animals. 2020. 10.3390/ani10101925. 33092108 10.3390/ani10101925 PMC7589328

[98] Ulutas B, Tan T, Ulutas PA, Bayramli G. Haptoglobin and serum amyloid a responses in cattle persistently infected with bovine viral diarrhea virus. Acta Sci Vet. 2011;39(3):973.

[99] Mohammadi S, Mohammadi V, Esmaeilnejad B. Evaluation of some acute phase proteins in cattle naturally infected with *Babesia**bigemina*. Comp Immunol Microbiol Infect Dis. 2021. 10.1016/j.cimid.2021.101642. 33735746 10.1016/j.cimid.2021.101642

[100] Khalphallah A, Elsayed HK, Mottelib AA, Mohammed MG. Ultrasonography as a differential diagnostic tool of bovine respiratory tract disorders with reference to serum haptoglobin and lipid profiles changes. J Adv Vet Res. 2022;12:153–65.

[101] Brodzki P, Marczuk J, Lisiecka U, Szczubial M, Brodzki A, Gorzkoś H, et al. Comparative evaluation of cytokine and acute-phase protein concentrations in sera of dairy cows with subclinical and clinical ketosis as a different view of the causes of the disease. Vet World. 2021;14:1572–8. 34316205 10.14202/vetworld.2021.1572-1578 PMC8304428

[102] Nazifi S, Ansari-Lari M, Asadi-Fardaqi J, Rezaei M. The use of receiver operating characteristic (ROC) analysis to assess the diagnostic value of serum amyloid a, haptoglobin and fibrinogen in traumatic reticuloperitonitis in cattle. Vet J. 2009;182:315–9. 18783967 10.1016/j.tvjl.2008.07.002

[103] Safarchi R, Badiei A, Nadalian MG, Seifi HA. Assessing predictive value of clinical, hematological and acute phase proteins in single and complex mode for diagnosing dairy cow peritonitis. Eur J Zool Res. 2014;3:38–48.

[104] Aydin O, Ulas N, Genc A, Baysal S, Kandemir O, Aktas MS. Investigation of hemogram, oxidative stress, and some inflammatory marker levels in neonatal calves with *Escherichia**coli* and coronavirus diarrhea. Microb Pathog. 2022. 10.1016/j.micpath.2022.105802. 36191841 10.1016/j.micpath.2022.105802

[105] Pirkkalainen H, Talvio I, Kujala-Wirth M, Soveri T, Orro T. Acute phase response of sole ulcer, white line disease and digital dermatitis in dairy cows. Vet Anim Sci. 2022. 10.1016/j.vas.2022.100253. 35669246 10.1016/j.vas.2022.100253 PMC9163099

[106] Donat K, Erhardt G, Soschinka A, Brandt HR. Decreased serum protein associated with *Mycobacterium**avium* subspecies *paratuberculosis* shedding in German holstein cows. Vet Rec. 2014. 10.1136/vr.101957. 24578317 10.1136/vr.101957

[107] Körmendy B, Szilágyi M, Tuboly S, Nagy G. Some diagnostic features of the pathogenesis of bovine paratuberculosis (Johne’s Disease) and serum biochemical changes after oral reinfection. J Vet Med. 1990;37:229–35. 10.1111/j.1439-0450.1990.tb01051.x 2385984

[108] Horadagoda NU, Knox KM, Gibbs HA, Reid SW, Horadagoda A, Edwards SE, et al. Acute phase proteins in cattle: discrimination between acute and chronic inflammation. Vet Rec. 1999;144:437–41. 10343375 10.1136/vr.144.16.437

[109] Alsemgeest SPM, Kalsbeek HC, Wensing T, Koeman JP, Van Ederen AM, Gruys E. Concentrations of serum amyloid-A (SAA) and haptoglobin (HP) as parameters of inflammatory diseases in cattle. Vet Q. 1994;16:21–3. 8009814 10.1080/01652176.1994.9694410

[110] Nazifi S, Ansari-Lari M, Tabandeh MR, Badiei K, Ghafari N, Karachi I, et al. Clinical relevance of serum sialic acids evaluation and correlation with haptoglobin and serum amyloid a in diseased cattle. Bulg J Vet Med. 2010;13:45–54.

[111] Choi KS, Kang JH, Cho HC, Yu DH, Park J. Changes in serum protein electrophoresis profiles and acute phase proteins in calves with diarrhea. Can J Vet Res. 2021;85:45–50. 33390652 PMC7747664

[112] Chae JB, Ku JY, Park KM, Choi KS, Chae JS, Park J. Hematology, serum biochemistry, and acute phase proteins in Hanwoo (Bos taurus coreanae) calves with diarrhea. J Vet Clin. 2022;39:342–52. 10.17555/jvc.2022.39.6.342

[113] Jaramillo C, Renaud DL, Arroyo LG, Kenney DG, Gamsjaeger L, Gomez DE. Serum haptoglobin concentration and liver enzyme activity as indicators of systemic inflammatory response syndrome and survival of sick calves. J Vet Intern Med. 2022;36:812–9. 35040515 10.1111/jvim.16357 PMC8965222

[114] Chen HH, Lin JH, Fung HP, Ho LL, Yang PC, Lee WC, et al. Serum acute phase proteins and swine health status. Can J Vet Res. 2003;67:283–90. 14620865 PMC280713

[115] Eckersall PD, Young FJ, McComb C, Hogarth CJ, Safi S, Weber A, et al. Acute phase proteins in serum and milk from dairy cows with clinical mastitis. Vet Rec. 2001;148:35–41. 11202551 10.1136/vr.148.2.35

[116] El-Deeb W, Fayez M, Alhumam N, Elsohaby I, Quadri SA, Mkrtchyan H. The effect of staphylococcal mastitis including resistant strains on serum procalcitonin, neopterin, acute phase response and stress biomarkers in Holstein dairy cows. PeerJ. 2021. 10.7717/peerj.11511. 34131523 10.7717/peerj.11511 PMC8174151

[117] Kamr A, Hassan H, Toribio R, Anis A, Nayel M, Arbaga A. Oxidative stress, biochemical, and histopathological changes associated with acute lumpy skin disease in cattle. Vet World. 2022;15:1916–23. 36313851 10.14202/vetworld.2022.1916-1923 PMC9615490

